# A Year of Infection in the Intensive Care Unit: Prospective Whole Genome Sequencing of Bacterial Clinical Isolates Reveals Cryptic Transmissions and Novel Microbiota

**DOI:** 10.1371/journal.pgen.1005413

**Published:** 2015-07-31

**Authors:** David J. Roach, Joshua N. Burton, Choli Lee, Bethany Stackhouse, Susan M. Butler-Wu, Brad T. Cookson, Jay Shendure, Stephen J. Salipante

**Affiliations:** 1 Department of Genome Sciences, University of Washington, Seattle, Washington, United States of America; 2 Department of Laboratory Medicine, University of Washington, Seattle, Washington, United States of America; 3 Department of Microbiology, University of Washington, Seattle, Washington, United States of America; Uppsala University, SWEDEN

## Abstract

Bacterial whole genome sequencing holds promise as a disruptive technology in clinical microbiology, but it has not yet been applied systematically or comprehensively within a clinical context. Here, over the course of one year, we performed prospective collection and whole genome sequencing of nearly all bacterial isolates obtained from a tertiary care hospital’s intensive care units (ICUs). This unbiased collection of 1,229 bacterial genomes from 391 patients enables detailed exploration of several features of clinical pathogens. A sizable fraction of isolates identified as clinically relevant corresponded to previously undescribed species: 12% of isolates assigned a species-level classification by conventional methods actually qualified as distinct, novel genomospecies on the basis of genomic similarity. Pan-genome analysis of the most frequently encountered pathogens in the collection revealed substantial variation in pan-genome size (1,420 to 20,432 genes) and the rate of gene discovery (1 to 152 genes per isolate sequenced). Surprisingly, although potential nosocomial transmission of actively surveilled pathogens was rare, 8.7% of isolates belonged to genomically related clonal lineages that were present among multiple patients, usually with overlapping hospital admissions, and were associated with clinically significant infection in 62% of patients from which they were recovered. Multi-patient clonal lineages were particularly evident in the neonatal care unit, where seven separate *Staphylococcus epidermidis* clonal lineages were identified, including one lineage associated with bacteremia in 5/9 neonates. Our study highlights key differences in the information made available by conventional microbiological practices versus whole genome sequencing, and motivates the further integration of microbial genome sequencing into routine clinical care.

## Author Summary

Bacterial whole genome sequencing is becoming increasingly common to microbiological research, but despite its great potential, has not yet been meaningfully integrated into clinical care. Here, we generated whole genome sequencing data from nearly all of the bacterial isolates prospectively collected from a hospital’s intensive care units over an entire year. Our analysis identifies novel microbiota in hospitalized patients, a high incidence of patient infection with multiple unrelated lineages of a bacterial species, and the possibility of cryptic transmission of bacteria among patients. Our study is unprecedented in providing a broad and unbiased view of bacterial infections that affect the hospital’s sickest patients, and demonstrates the extent of information that can be learned from comprehensive genomic surveillance of clinical bacterial isolates over an extended period of time.

## Introduction

Primary and nosocomial bacterial infections are a major source of morbidity and mortality worldwide [1]. In the United States alone, community-acquired bacterial infections represent a leading cause of death, particularly among the elderly [2,3], while healthcare-associated bacterial disease affects approximately 2 million patients annually and results in nearly 100,000 fatalities [4,5]. Bacterial infectious disease is especially pervasive among patients undergoing intensive medical care, who are predisposed to disease because they are frequently immuno-compromised [6], have undergone invasive procedures, and/or have long-standing placement of foreign bodies (such as indwelling catheters and mechanical ventilators) [7]. Furthermore, effective treatment of bacterial infections has been complicated by the emergence and dissemination of multi-drug resistant strains [8–10], which can colonize healthcare environments and persist in them over time [11,12].

Although predominantly restricted to research studies at present, bacterial genome sequencing of clinical isolates is becoming increasingly tractable as the cost of DNA sequencing continues to decline [13,14]. Bacterial genome sequencing has shed light on diverse aspects of a number of medically-relevant pathogens, including global and intra-hospital strain transmission [9,15–19], the evolution of antibiotic resistance [15,20,21], horizontal exchange of antibiotic resistance plasmids within hospitals [11], the distribution of genomic material across a bacterial species [22,23], and the identification of novel bacterial pathogens [24]. Although such studies have provided important insights and an improved understanding of medically important bacterial pathogens, in general, nearly all have been limited in one or more respects: they have been retrospective in nature, limited in scope to particular bacterial species, and/or have extended to a subset of bacterial isolates (e.g. a recognized outbreak), rather than globally across an entire patient population. These limitations impose an artificially narrow and biased perspective on the repertoire of infectious organisms affecting patient populations [25].

As a different approach, we sought to perform prospective collection and whole genome sequencing of all bacterial isolates obtained from the intensive care units of a single tertiary care hospital over a period of one year. This collection of 1,229 microbial genomes enables quantification of several features of infectious bacteria within the patient population of a single hospital, including genetic diversity, the prevalence of previously unreported bacterial species, and large-scale, longitudinal reconstruction of both intra-patient and inter-patient molecular epidemiology.

## Results

### Specimens

This study was conducted from August 2012 to August 2013 at the University of Washington Medical Center, a 450-bed hospital in Seattle, Washington, that serves as a tertiary care center for a geographic region comprising 5 states. Bacterial isolates were obtained during the course of routine medical care of patients in the hospital’s three intensive care units (neonatal, medical/surgical, and cardiac). All individually characterized bacterial isolates (i.e, isolates receiving a species-level, genus-level, or descriptive classification) that were reported in patients’ laboratory results were collected for sequencing.

A total of 1,316 viable isolates obtained from 421 individual patients were subjected to shotgun whole genome sequencing. Libraries from 87 isolates failed quality control, leaving in a final collection of 1,229 isolates obtained from 391 different patients (222 males and 169 females) (**S1 Data File**). The average age of patients in the neonatal intensive care unit was 34 days at the time of sampling (range 0–109 days), while the average age of patients occupying other units was 56.6 years (range 17–94). The sequenced isolates originated from a variety of anatomic locations, although cultures of broncheoalveolar lavage fluid, sputum, blood, urine, and wounds collectively accounted for 80% of sampling sites (**S1 Fig**). Because sampling locations included normally non-sterile sites, in some cases our collection represented both known bacterial pathogens and normal microbiota sampled incidentally. In accordance with the laboratory’s testing practices, we considered an isolate to be clinically relevant if there was an indication necessitating antibiotic susceptibility profiling, either as part of the clinical laboratory’s reflex testing algorithms or by special physician request.

### Genomic classification and identification of novel genomospecies

**Fig 1:**
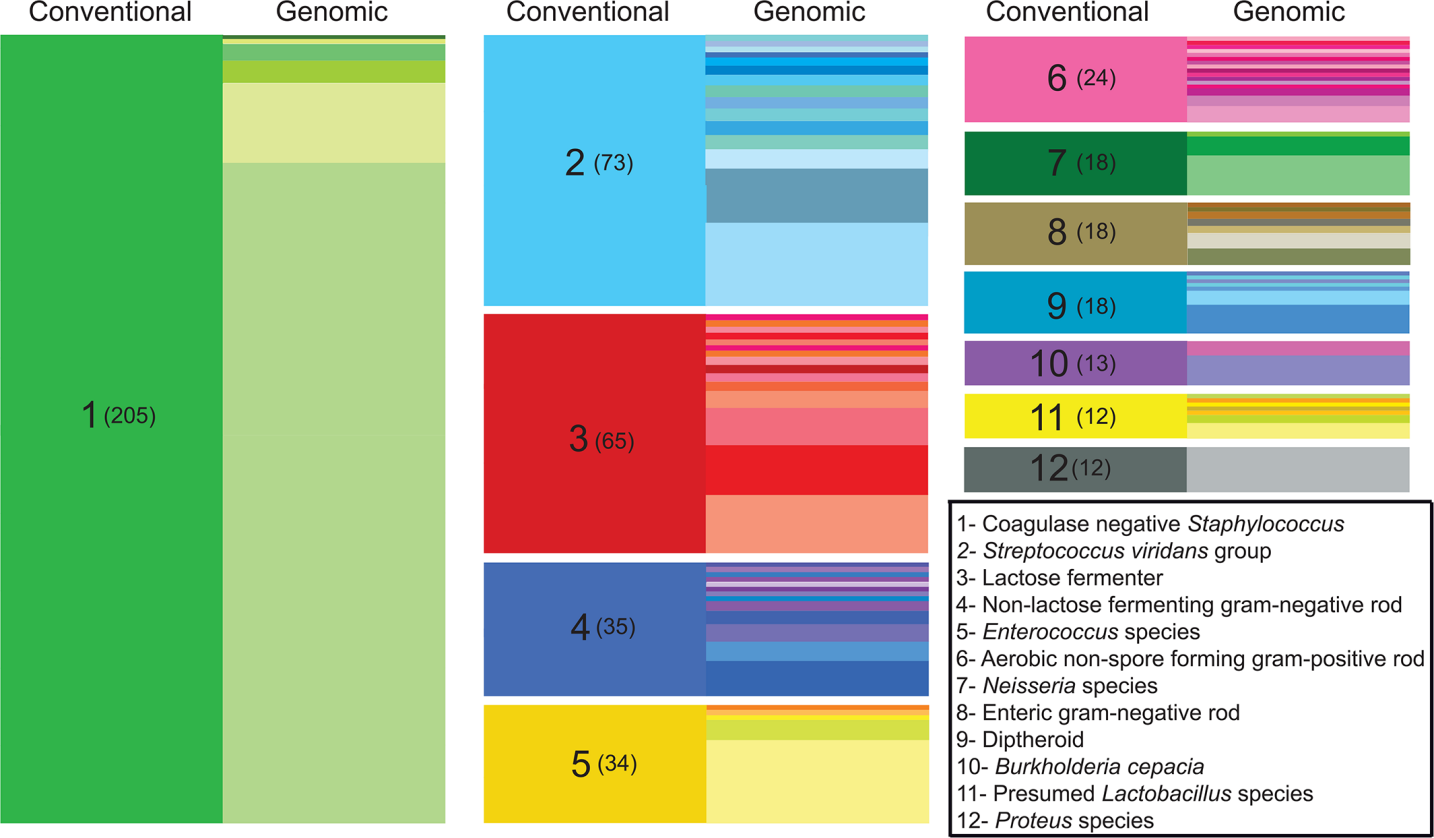
Improved taxonomic resolution of bacterial typing by genomic similarity. Data are shown for the 12 most frequent non-species-level classifications assigned by the clinical laboratory. These classifications encompassed 43% (527/1,229) of the isolates in our sample set, while isolates given a species-level classification by the clinical lab accounted for nearly 50% (612/1,229 isolates). On the left side of each column (“conventional”), colored blocks represent a classification assigned by the clinical microbiology laboratory based on phenotypic and/or biochemical characterization. The clinical classification and the number of isolates receiving the classification (in parentheses) are shown in the left column of each block. In the right side of each column (“genomic”), differently colored partitions indicate individual species-level assignments based on genomic similarity (closest identity to a representative of the species) for isolates of with the indicated clinical classification. The height of each colored partition in a column is proportional to the number of isolates it represents.

Conventional clinical microbiology workup resulted in a species-level classification for nearly half of the bacterial isolates (610/1,229), the majority of which corresponded to well-characterized bacteria such as *Escherichia coli* and *Staphylococcus aureus*. The remaining isolates were classified at the genus level (ie, *Lactobacillus sp*.), or on the basis of morphological and/or metabolic characteristics (for example, “lactose-fermenting gram negative rod”), according to recommended practice guidelines [26]. Altogether, the harvested organisms encompassed 83 distinct taxonomical or functional classifications (**S2 Data File**) as determined by routine clinical laboratory testing. To enable more precise taxonomic classification of isolates sampled from the clinical wards, we performed *de novo* genome assembly for each isolate and identified the closest matching (partial or complete) genome present in the NCBI nucleotide database. Using this strategy, we identified 126 different species from 60 different genera (**S2 Data File**), providing a highly resolved catalog of the organisms collected from the ICU system (**Fig 1**). For many clinical classifications not extending to the species-level, it is perhaps unsurprising that a genomic approach readily uncovers heterogeneity not represented through existing bacterial typing algorithms, and more definitively establishes organism identity.

Because our isolates were derived from otherwise unselected primary clinical material, we hypothesized that our collection could contain organisms, potentially including medically significant organisms, which are not well represented in existing curated repositories. In order to explore the prevalence that novel species were represented within the primary clinical isolates, we calculated pairwise average nucleotide identity by BLAST (ANIb) [27] between each isolate and its closest identified match from NCBI. Pairwise ANIb values of less than 95% are generally accepted as the cutoff for circumscribing separate species [27,28]. Species demarcations based on this definition of genomic similarity are known by the technical definition of ‘genomospecies’, indicating that they have not been established on the basis of identifying phenotypic or morphological characteristics. Pairwise ANIb values for our isolate collection ranged from 71–100%, with 35% (428/1,229) of all isolates falling below a 95% identity threshold to its closest matched genome, and thereby qualifying as novel genomospecies (**S2 Fig**). Such organisms were isolated from all culture sites (**S1 Fig**), suggesting that they were not restricted to heavily colonized anatomic locations such as the gut and skin. Although none of the classifications by the clinical laboratory were overtly different from the closest-matched genome identified for any given isolate, 12% (76/612) of isolates receiving a species-level classification displayed <95% ANIb to the specified taxon, and thus represented a genomospecies distinct from the given clinical classification. Novel genomospecies were unevenly distributed across bacterial genera; at the extremes, all isolates from the genera *Escherichia*, *Lactobacillus*, and *Proteus* were grouped with a previously reported genome, whereas all isolates from the genera *Neisseria* and *Corynebacterium* fell below a 95% ANIb identity to previously reported genomes (**S3 Fig**), likely reflecting the high degree of genomic diversity inherent to those lineages [29,30].

**Fig 2:**
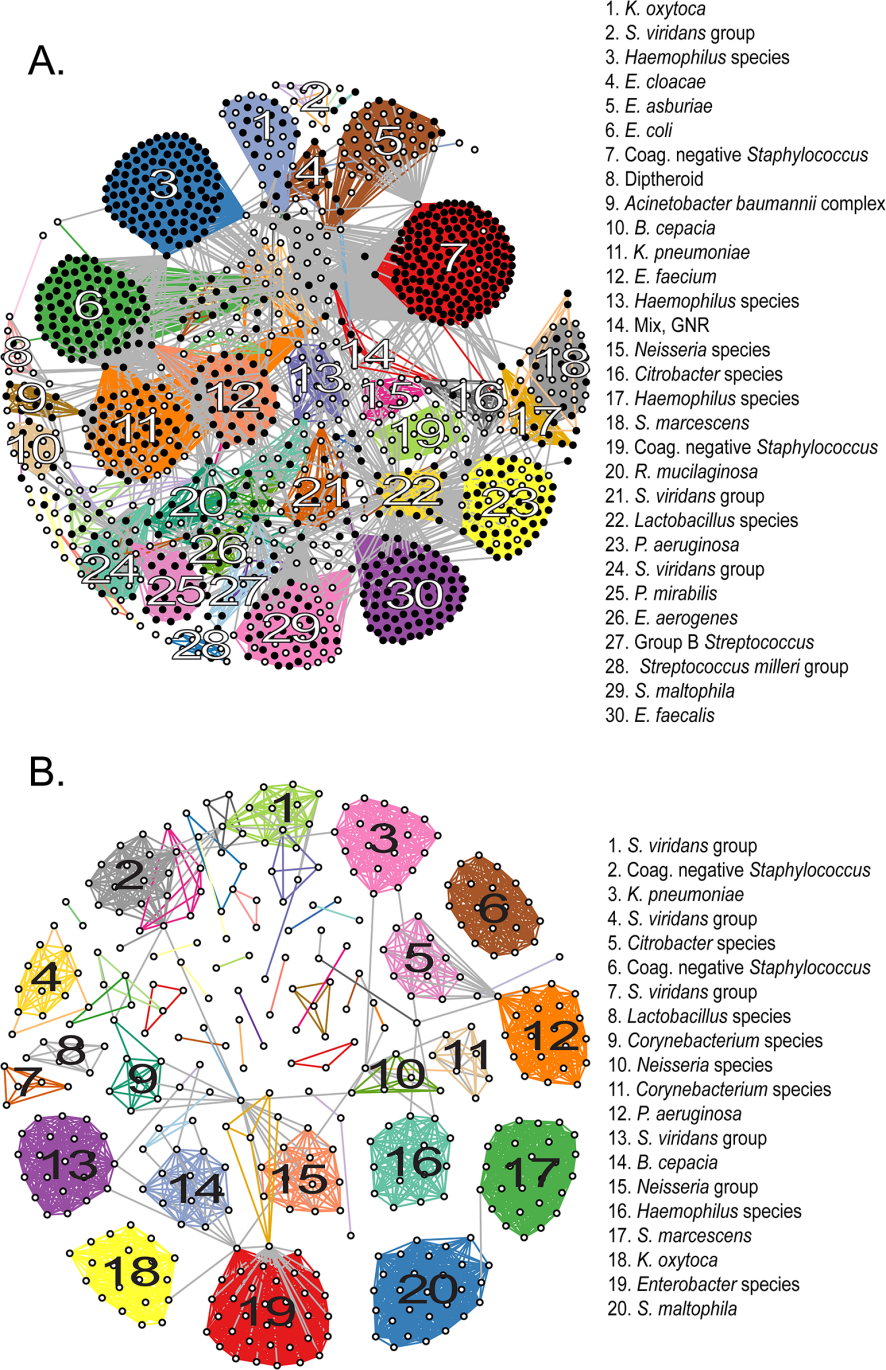
Clustering of sequenced isolates by genomic similarity. (**A**) Network diagram of all 1,229 sequenced isolates that could be assigned to one of 78 clusters on the basis of pairwise ANIb. Each node represents an individual isolate, and is colored black if robustly matching a previously reported genome (≥ 95% ANIb) or white if corresponding to a novel genomospecies. Nodes connected by a visible edge indicate pairwise ANIb values ≥ 95%. Edges connecting isolates within the same cluster are colored according to that cluster, edges connecting isolates that match multiple clusters are grey. Clusters are labeled according to the most detailed taxonomic classification given to isolates during conventional identification. (**B**) Network diagram of 419 isolates corresponding to novel genomospecies, assigned to 53 clusters on the basis of pairwise ANIb. Clusters are labeled as in (A). For both panels, the length of edges between nodes is not informative or proportional to ANIb values, and consequently neither is the placement of specific nodes or groups within the graph. The amount of connectivity among nodes indicates the basis of their inclusion with respect to specific groups.

We next evaluated groups of novel genomospecies with respect to their genomic similarity to known organisms. We performed an all-by-all computation of pairwise ANIb values among sequenced isolates, built a graph from these data, and clustered like isolates in accordance with their pairwise ANIb scores (**Fig 2A**). The 1,229 isolates fell into a most likely configuration of 78 distinct groups (indicated by the maximal intra-cluster enrichment metric [31], **S4 Fig** and **S3 Data File**), defined by chains or clusters of 2 or more isolates linked with values of ≥95% pairwise ANIb, and an additional 29 isolates were left unclustered as singletons. This number of categories is somewhat less than the number of species obtained by conventional or genomic classification and suggests that our “*de novo*” clustering approach provides a more conservative estimate of the number of genomospecies (**S2 Data File**). Pairwise comparisons of isolates in the same group showed significantly (p< 0.0001, Mann-Whitney test) higher ANIb values than those in different groups (97% ANIb versus 43% ANIb, respectively), and a clear separation of these categories around the 95% ANIb threshold, supporting the validity of clustering results (**S6 Fig**).

Fourteen (18%) non-singleton clusters were comprised entirely of isolates classified to a previously described species, that is, they contained exclusively organisms with ≥95% ANIb to representatives of that species; 22 clusters (29%) contained ≥50% isolates matching a particular known species (with a minority of isolates qualifying as a novel but related genomospecies); in another 12 clusters (15%) the majority of isolates were novel genomospecies, with a minority assigned to known taxa. Finally, 30 clusters (39%) were made up entirely of isolates qualifying as novel genomospecies, although many of these (19/30) were composed of only two or three isolates each. 76% (22/29) of unclustered isolates were novel genomospecies.

In order to best quantify the number of novel genomospecies represented in the collection, we repeated clustering using only those isolates that did not match previously sequenced genomes with ≥95% ANIb (**Fig 2B**). These distributed among 53 separate clusters (**S5 Fig and S4 Data File**) and 34 singletons, conservatively suggesting that our survey has identified 87 novel taxonomic groups. Of those categorizations, 32% (28/87) contained at least one isolate with an indication for antibiotic susceptibility testing, and so harbored a fraction of isolates which satisfied our definition for clinical significance. Of note, 20 of these 28 groups contained two or more representatives obtained independently from more than one patient, corroborating their significance in multiple contexts and individuals.

### Genomic diversity of clinical isolates

Existing pan-genome analyses have not included large numbers of primary clinical isolates. To measure genomic diversity observed within established clinical pathogens, we assessed the pan-genome content of the 20 most prevalent species cultured from our patient population (range: 6 isolates for *Moraxella catarrhalis* to 162 isolates for *Staphylococcus epidermidis*). For each species, we considered the number of unique genes represented in existing reference genomes and subsequently factored in predicted genes from the sequenced clinical isolates. We restricted analysis to isolates with >95% ANIb to established reference genomes in order to avoid inflating these estimates.

**Fig 3:**
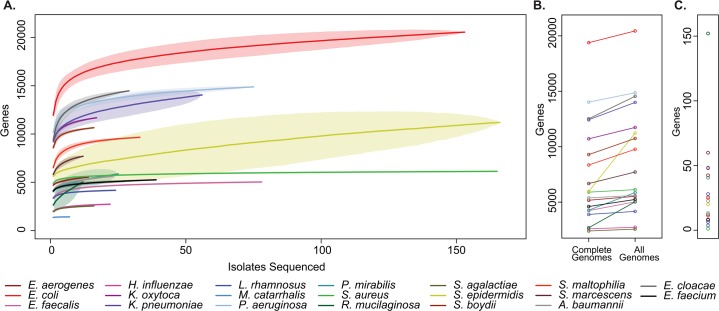
Pan-genome curves of the most abundant organisms recovered from clinical sampling. **(A)** The total number of non-orthologous genes comprising the pan-genome, after excluding prophages and insertion sequences, for the 20 most prevalent organisms collected. Pan-genome size is shown for the indicated number of analyzed genomes. Plots are derived from organisms’ available reference genomes and the isolates sequenced in this study. Mean values are plotted for each estimate by a solid line, standard deviation for estimates are indicated by lightly shaded regions. (**B**) Pan-genome size calculated only from existing reference genomes, and after incorporation of all isolates sequenced. (**C**) Current rate of gene discovery, considering reference genomes and newly sequenced clinical strains.

For all bacterial species examined we added additional genes to the pan-genome with each clinical isolate sequenced (**Fig 3A** and **S5 Data File**). This analysis indicates that common clinical pathogens all display “open” pan-genomes with versatility in gene content [32], albeit to varying degrees. Relatedly, across taxa we consistently identified additional unique sequences not represented in reference genomes, indicating that even in well-studied pathogens, there remains a reservoir of uncatalogued genomic variation harbored by clinical isolates (**Fig 3B**).

Considering the collection as a whole, there was substantial variation between species with respect to the overall size of the pan-genome, the number of unique sequences that were discovered outside of existing reference genomes, and the current rate of gene discovery (**Fig 3C**). There was a weak relationship between the number of isolates sequenced and the rate of gene discovery (R^2^ = 0.129). Perhaps unsurprisingly [33], *Escherichia coli* had the largest pan-genome size of the species analyzed with 20,432 non-homologous genes, while *Moraxella catarrhalis* contained the fewest, with 1,420. At the conclusion of this study, new genes were discovered in *Rothia mucilaginosa* at a rate of 152 per additional isolate sequenced (11 clinical isolates sequenced), making its pan-genome the least explored of the organisms included in our sample set. At the other extreme, only 1 new gene was discovered with each additional *S*. *aureus* isolate sequenced (108 clinical isolates sequenced), suggesting that there is relatively little unexplored distributed genetic content in our collection.

### Isolate clonality and molecular epidemiology

We explored the molecular epidemiology of bacterial infections within the hospital, investigating both the possibility of bacterial transmission events and the dynamics of infection within individual patients. To ensure sufficient genomic similarity for accurate mapping and variant calling, we again restricted analysis to isolates with >95% ANIb to established reference genomes.

Initially, we sought to quantify the level of artifactual genomic variation arising from our protocols. We randomly selected sixteen isolates distributed across 15 different species, and for each, paired technical replicates were separately taken through library construction, sequencing, and analysis. No discordant single nucleotide variants (SNVs) were identified between any replicate pair.

Because strains may undergo genomic diversification during the course of an epidemic [34–36], or even during infection in a single patient [37–39], establishing a quantitative definition for the number of genomic polymorphisms which identify isolates as members of the same outbreak remains an unresolved challenge in molecular epidemiology [34]. Indeed, accurately reconstructing transmission chains in light of this so-called “cloud of diversity” will require defining new practices in molecular epidemiology, including examination of multiple isolates from the same individual [35,39]. Given these issues, here we defined strain clonality (relationship by direct descent) using two different thresholds: A] low stringency: isolates distinguished by up to 40 SNVs, a threshold based on empiric measurements of within-host bacterial genomic variability [40], and B] high stringency: isolates distinguished by no more than 3 SNVs [41], based on the variability of genomic sequencing in other studies. It is expected that the low stringency threshold encompasses potentially indirect transmission events, such as those occurring through intermediate reservoirs such as the environment or asymptomatic colonized hosts, where lineages have had time to accumulate some number of differentiating genomic differences [39,40], whereas the high stringency threshold is more likely to represent direct transmission of bacterial clones [41]. A key point is that both of these cutoffs fall vastly below the average pairwise distance that is expected between any two randomly selected isolates of the same species from the larger community (**S1 Table**). We considered isolates to be members of the same clonal lineage if they were linked either directly or through intermediate connections of equal to or less than these thresholds.

#### Intra-patient infection dynamics

To explore the properties of bacterial infection at the level of individual patients, we performed two related lines of analysis. First, for all patients, we examined whether infection with a particular agent could be attributed to a single clone or to polyclonal infection (infection caused by multiple strains of the same species). Second, for patients with persistent recovery of the same bacterial species longitudinally, we looked for changes in the makeup of infectious lineages over time.

To examine the incidence of polyclonal infection, we first identified specimens where at least 2 isolates belonging to the same species were recovered from a patient within 20 days of one another, and found a total of 135 cases of multiple isolates (obtained from 108 patients) that met this requirement. In 32 cases involving 24 different patients, isolates displayed >40 SNVs, and thus could be considered polyclonal. Nine of these cases represented isolates sampled from disparate anatomic compartments, and we could therefore not rule out representation of distinct colonization or infection events. However, genomically distinct isolates in 23 cases were sampled from the same culture site, with 18 meeting our functional definition of clinical significance and thereby qualifying as polyclonal infections (**S2 Table**). Nevertheless, in 11 instances only one representative of the species was subjected to antimicrobial susceptibility testing, potentially missing phenotypic differences in unrelated strains that were not tested. The most commonly polyclonal species was *S*. *epidermidis*, with 14 instances of polyclonality identified, including 4 cases of polyclonal bacteremia. The average number of pairwise differences between *S*. *epidermidis* lineages in these polyclonal infections was 5,514 SNPs (range = 46 to 20,704 differences, standard deviation = 4,432 differences), suggesting the presence of highly diverged *S*. *epidermidis* strains within the same patient. Furthermore, *S*. *epidermidis* was the only species to have more than 2 genomically distinct strains simultaneously recovered from a patient: four patient specimens each had 3 distinct strains isolated concurrently, while two specimens demonstrated 4 different lineages each.

To examine the clonality of infections over time, we next examined 32 patient cases where the same bacterial species was recovered from a patient longitudinally over a period of 20 days or more (**S2 Table**). In 15 patients, isolates were distinguishable at the genomic level (>40 SNVs), suggesting multiple inoculations with distinct lineages or persistent polyclonal infection. In the other 17 cases, isolates comprised members of the same clonal lineage that were recurrently cultured from the same patient (10 different bacterial species, time between samplings ranging from 22 to 105 days, average 26.7, standard deviation 26.3 days). These repeat samples often occurred from distinct anatomic locations (9/17 patients), suggesting greater anatomic distribution of the clone. In 10 instances, patients received antibiotic therapy for the infection, but the same clonal lineage was nevertheless recovered at a later time. Furthermore, in most cases (14/17), clonal lineages displayed fewer than three distinguishing variants between any two isolates in the same complex, meeting our stringent definition of clonality (**S7 Fig**). There were two species wherein intrapatient clonal lineages only met the low stringency definition of clonality: the two longitudinally-sampled *S*. *aureus* clonal lineages and the one *Klebsiella pneumoniae* clonal lineage. Notably, in the case of the *K*. *pneumoniae* complex a patient received antibiotic therapy after the first isolate was cultured from a broncheoalveolar lavage sample, and 105 days later, a *K*. *pneumoniae* isolate with 7 distinguishing variants from the original was cultured from the patient’s urine during workup of a urinary tract infection. This anecdotal example suggests both a failure of therapy to completely eliminate the pathogen and re-infection of anatomically distinct body compartments.

#### Evidence for inter-patient transmission events

**Fig 4:**
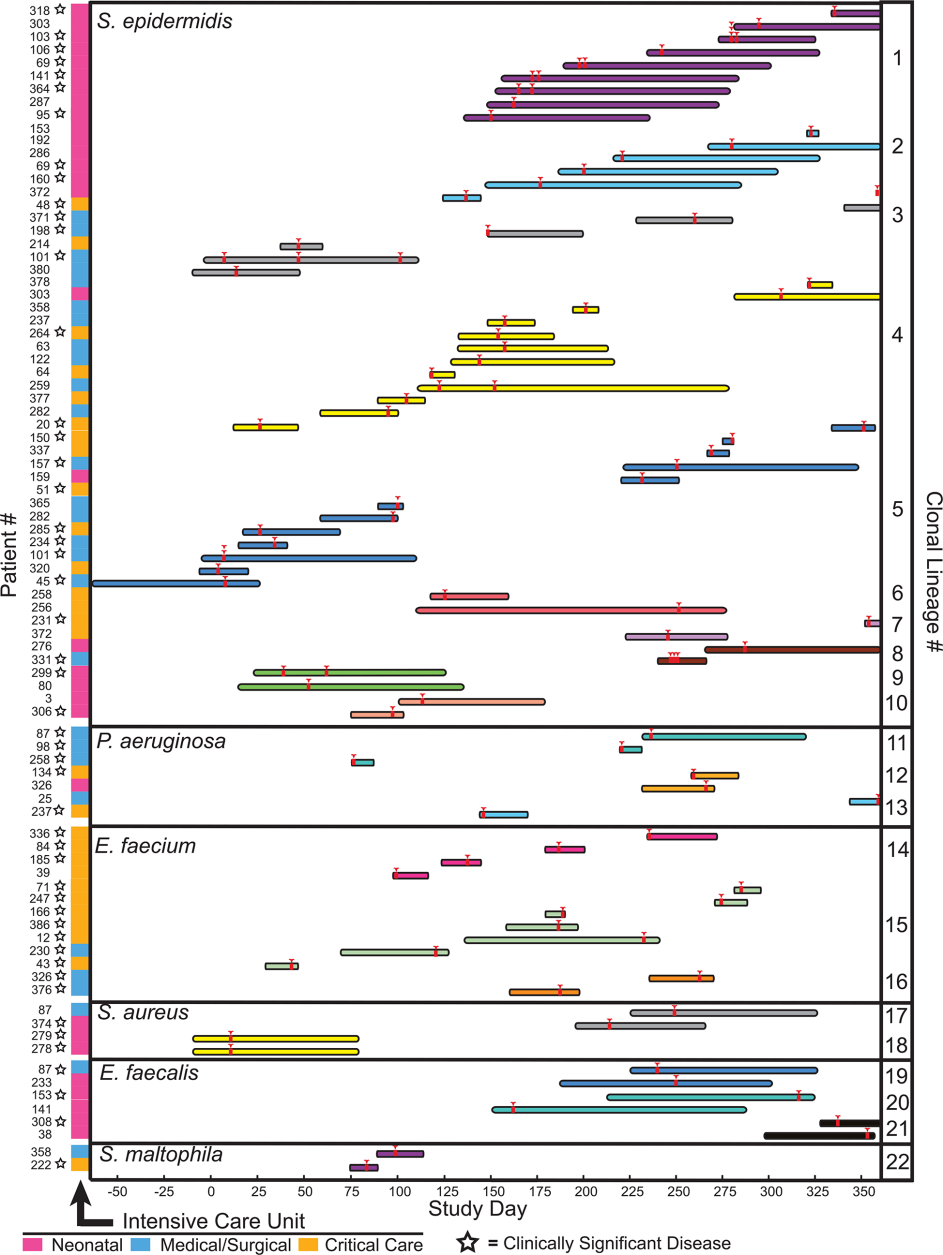
Clonal lineages extending across multiple patients. Clonal lineages are stratified by the indicated organisms. The x-axis indicates the timeline of this study, with day 0 corresponding to the first day of sample collection. Infection events in specific patients are represented by individual rows, with the length of the bar indicating the dates of a patient’s hospitalization. Bars are colored and grouped according to clonal lineages, and red arrowheads denote specimen collections where an isolate in the clonal lineage was obtained. The patient number and the hospital ward where the patient was receiving treatment when culture was performed is shown at the left (ICU legend below), and stars denote isolates that were associated with clinically significant infection (infections therapeutically targeted by physicians).

Isolates from clonal lineages spanning multiple patients, and therefore reflecting possible inter-patient transmission (or acquisition) events, were recovered 89 separate times, involving 22 distinct clones from 6 different bacterial species, and usually in the context of overlapping hospital admissions (**S3 Table** and **Fig 4**). Clonal isolates were recovered from 18% (71/391) of patients and accounted for 8.7% (108/1,229) of the bacteria in our sample set. Collectively, isolates from clonal lineages were associated with clinically significant infection, and were treated with antibiotic therapy, in 62% (44/71) of the patients from whom they were recovered, as ascertained by chart review.

The majority of clonal lineages involved in acquisition events (14/22) comprised a single clone isolated from two separate patients (**Fig 4,** clonal lineages 6–10,12–13, and 16–22). 6 of those acquisitions involved isolates with three or fewer distinguishing variants. In one instance (clonal lineage 18), twin newborns each presented with *Staphylococcus aureus* bacterial pneumonia, and isolates differing by 4 SNVs were cultured from them. This example likely reflects common inoculation from maternal microbiota [42] rather than nosocomial transmission.

We also identified larger-scale clonal lineages, involving 3 or more patients infected with a clonal lineage of *Staphylococcus epidermidis*, *Pseudomonas aeruginosa*, or *Enterococcus faecium* (**S6 Data File**).

*S*. *epidermidis* clonal lineage acquisitions involving more than two patients (clonal lineages 1–5) were the most frequently identified among patients, demonstrating both the greatest number of events (46) and the largest number of distinct clones (5). Review of medical records indicated that all five *S*. *epidermidis* clonal lineages were associated with clinically significant infection, ranging from wound infections to bacteremia, and required medical intervention in 22 different patients. Strikingly, *S*. *epidermidis* isolates from four different large-scale clonal groups were detected in the neonatal intensive care unit. Clonal lineages 1 and 2 were most prevalent, being cultured from 9 and 6 infants, respectively, and were repeatedly recovered from patients on the unit over the course of 229 days. Clonal lineage 1 was associated with sepsis in 5/9 neonates from whom it was isolated, suggesting a particularly virulent strain. Isolates from that complex were either genomically indistinguishable (no pairwise variants detected) or were closely related, with a range of 0–18 SNVs between any two (average 6.9, standard deviation 4.5 variants) (**S6 Data File**). This level of genomic identity suggests a recent common ancestry.

Inferred transmission of *P*. *aeruginosa* and *E*. *faecium* clones was not as extensive as *S*. *epidermidis*, but tended to be associated with a more insidious clinical course.

The *P*. *aeruginosa* clonal lineage (clonal lineage 11) was a multi-drug resistant strain isolated from three different patients. The complex demonstrated a moderate level of genomic dissimilarity (pairwise differences of 13, 23 and 25 SNVs). On further analysis, this lineage was found to carry missense mutations in *mutS*, *mutL*, and *uvrD* genes (**S4 Table**), suggesting a hypermutator strain [43], and a potential cause of the heightened genomic diversity in this clonal lineage. The first isolate sampled from this clonal lineage was cultured from the blood of a patient admitted from an outside facility with *Pseudomonas* sepsis and who was discharged after undergoing successful antibiotic treatment. Almost five months later, *P*. *aeruginosa* isolates were grown from the blood cultures of two different patients undergoing treatment in the same unit and whose hospital stays overlapped by one day, and the isolates from those patients differed from one another by 13 SNVs. All three isolates in the clonal lineage had an identical antibiotic resistance profile, and each was the implicated agent of bacteremia in its patient of origin. One patient died from their infection.

The two *E*. *faecium* clonal lineages (clonal lineages 14 and 15) appeared similarly infectious, causing disease requiring clinical treatment in 10/11 patients, and they shared an identical drug resistance profile that included resistance to vancomycin. The isolates of the larger *E*. *faecium* clonal lineage (complex 8) demonstrated a range of 2–48 pairwise SNVs (average 29.6, standard deviation 12.6 variants) and caused infection of blood, wound, abdominal fluid, or the urinary tract in all 7 patients from whom it was cultured. The smaller *E*. *faecium* complex (complex 7) displayed a similar level of genomic variation (range 7–41, average 26.7, standard deviation 13.6 variants) but a more mild clinical course; it was incidentally isolated from the sputum cultures of one patient and from the urine of three patients with UTIs secondary to catheterization. *E*. *faecium* isolates from both clonal lineages were cultured throughout the year of sample collection, suggesting persistence of the clone within the hospital environment and consistent with reported patterns of vancomycin resistant *Enterococcus* in other hospitals [10].

## Discussion

It has been posited that large-scale, whole genome sequencing of clinical bacterial isolates holds promise as a transformative technology in the practice of clinical microbiology [9,13,44,45] but the technology has not yet been systematically applied in a clinical context to explore what information can be readily obtained from using this approach. In one noteworthy study, all 130 bacterial isolates recovered from a clinical microbiology laboratory over a single day were subjected to whole genome sequencing [46], although the analytic scope of that effort was limited to taxonomical classification of the organisms. Other, recent work has explored the prospective [19] and “real-time” [47] use of bacterial whole genome sequencing to define the molecular epidemiology of suspected disease outbreaks. However, to the best of our knowledge, our study is unique in that we prospectively performed large-scale, unbiased collection of all bacterial isolates from a defined set of hospital units over an extended time period and performed genomic sequencing and analysis. Consequently, this project reveals a number of telling differences between the information obtained by existing microbiological practices and what can be learned from genomic analysis of clinical bacterial isolates.

First of these is the straightforward task of classifying an isolate within the bacterial taxonomy. In standard clinical practice, taxonomic classification of bacterial species has long relied on phenotypic characteristics such as Gram stain, colony morphology, and biochemical characterization. The degree to which particular isolates from a specimen are assigned a species-level classification is designed, in part, to avoid reporting members of the normal microbiota or other organisms that are non-contributory to a disease process, and thus discouraging antibiotic “overtreatment” by physicians [26,48]. The reporting of species-level classifications therefore depends on the availability of microbiological methods for classifying an organism, the clinical indication for culture, the anatomic sampling site, and special requests of the ordering provider. Consequently, bacterial isolates from a specimen are neither typed comprehensively nor all typed to the same level of taxonomic resolution, and some species-level identifications made in the clinical laboratory are not reported to providers. Although the current utility to clinical practice is debatable, with whole genome sequencing it is possible to readily and unilaterally classify isolates on the basis of sequence similarity to other reported draft or reference genomes, providing improved granularity and eliminating ambiguities presented by genera or group-level assignments (i.e., “Coagulase negative *Staphylococcus*”). Although no overt inconsistencies were observed between isolates’ genomic classifications and conventional taxonomic assignment, whole genome sequencing provided substantially greater taxonomic resolution than standard practices (**Fig 1**). Additionally, a sizable fraction of all isolates (35%) corresponded to novel genomospecies upon genomic analysis, including 12% of the isolates that were assigned a species-level taxonomic classification by conventional methods. Although somewhat surprising, this finding is compatible with earlier, 16S rRNA-based typing studies wherein a significant fraction of infections were caused by previously uncharacterized organisms [49] that conventional microbiology practices are incapable of resolving. Further characterizing these organisms and exploring their prevalence and potential pathogenicity will be important work for future studies.

The flexible genome content of bacteria is a major contributor to their pathogenicity [14,22,50], and investigation of the pan-genomic content of a species can therefore provide insights into aspects of biology relevant to pathogenesis. All pan-genomes examined in this study (**Fig 3**) increased predictably with additional isolates sequenced, suggesting that additional genomic content will be identified with continued sequencing of primary clinical isolates. Further, these results suggest that the availability of high quality reference genomes is not necessary to explore genomic content and to identify potentially important genes relevant to human disease. Properties of pan-genomes varied considerably across species, particularly with respect to the rate of gene discovery per additional strain sequenced. *Staphylococcus aureus* clinical isolates in our collection had the lowest rate of novel gene discovery, suggesting that clinical lineages are unlikely to experience major phenotypic shifts owing to the acquisition of new functions coded by external genes. The finding of an essentially “closed” pan-genome for *S*. *aureus* strains is surprising in light of previous reports of extensive genomic variation for that species [51], and likely reflects the relative clonality of the predominant clinical strain [52]. In contrast, organisms such as *Klebsiella pneumoniae* and *E*. *coli* display genomic content that is broadly distributed across its members, signifying an “open” pan-genome [11,23]. The observation that pangenome size for those species does not dramatically plateau even with large numbers of additional strains sequenced supports the notion that their pangenomes are actively evolving [23,53][14], and that discoveries in additional genomic diversity may be sustained indefinitely.

With respect to intra-patient infection dynamics, we identified a measurable frequency of polyclonal bacterial infections (30.5% of isolates from a given species that were recovered from a patient within 20 days). However, since it is not clinical practice to isolate phenotypically indistinguishable isolates of the same species, this is almost certainly an underestimate secondary to incomplete representation in our isolate collection of all lineages involved in particular infections. Relatedly, although it is current microbiological practice to characterize the antibiotic profile of phenotypically distinct representatives of the same species (typically, those with different colony morphologies), our data indicate that in some cases typing multiple isolates from a particular species could yield significantly different results. In contrast, the majority (17/32) of persistent bacterial infections, recovered from a patient over 20 days or more, were marked by longitudinal recovery of the initial infecting clone. These findings suggest that many cases of long-lasting or chronic infection reflect continuous colonization of the original infectious strain, or autoinoculation from untreated bodily sites.

The most unanticipated results of our study are from molecular epidemiological analysis of bacterial clonal lineages, where we find data consistent with direct (0–3 SNV differences among isolates) and less direct (up to 40 SNV differences among isolates) bacterial transmissions involving multiple patients in these ICUs. The dynamics underlying bacterial colonization and transmission are surprisingly complex [34,39,40], and circumscribing transmission events according to specific thresholds of strain relatedness is certainly an oversimplification. For example, this strategy could exclude transmission of hypermutator strains separated by an unusually high mutational burden [54,55], or those spread by individuals over the course of long-term carriage [39]. In this respect, our analysis may be considered somewhat conservative, but is appropriately restrictive given the unbiased sampling employed in our study.

Though the importance of nosocomial transmission of bacteria has long been a major public health concern [5,56], whole genome sequencing studies by several groups have recently shown that the true incidence of nosocomial transmission is quite rare for several common pathogens of major concern [14,16,18,57,58]. Our findings support the conclusions of those studies when overlapping the specific pathogens that have been previously surveyed; however, evidence is provided in our study for the inter-patient sharing of several opportunistic pathogens: *S*. *epidermidis*, *E*. *faecium*, and *P*. *aeruginosa*, and to a lesser extent, *E*. *faecalis*, *S*. *aureus* and *S*. *maltophilia* (**Fig 4**). The most notable of these clonal lineages affected the NICU, and involved at least seven different *S*. *epidermidis* clonal lineages (3 involving pairs of patients and 4 spanning 3 or more patients). Because neonates are initially colonized with microbiota both from the maternal genital tract [42] and microbes from the external environment [59], they may be especially susceptible to colonization with transmitted agents compared to patients with more established microbiomes.

A substantial fraction of the observed clonal lineages were associated with bacterial disease that required clinical treatment (44/71 patients). However, the sharing of closely related strains among patients was not recognized nor further investigated in the absence of genomic data. There are several explanations for this. First, except in rare cases, bacterial characterization of coagulase negative *Staphylococcus* (other than *Staphylococcus lugdunensis*) was not performed to the species level, so that identification of recurrent *S*. *epidermidis* infections would not have been recognizable. Secondly, many of the clonal lineages were transmitted over extended periods of time (up to nearly a year) and in some cases without any temporal overlap of affected patients, making it difficult to link the cases on the basis of patient biogeographical information. All organisms involved are known to be transmitted by person-to-person interaction as well as via intermediate colonization of the hospital environment itself [10,17,58,60–63]. Thus, in addition to potential environmental reservoirs that remain unexplored in our study, it is possible that some of these events were mediated by assimilation of clonal lineages into the normal microbiota of individuals of the hospital staff without causing disease (“cryptic” or “silent” transmissions [9,17]), and inadvertently transmitted to patients over time through the circulation of medical staff among the different units. It is possible that key epidemiological links between individuals were not captured by, or represented in, our study. Third, the organisms involved all carry the distinction of being both commensal organisms and opportunistic pathogens, and consequently, autoinfection of a patient with their resident microbiota would be the most likely explanation for any isolated case.

We emphasize that, although our analysis reveals isolates that are likely to be related by descent, it is unclear how and when transmission of clonal lineages occurs. For example, it is possible that some transmissions reflect indirect infection or colonization events occurring outside the hospital from endemic, clonal bacterial pools present in the community [18,64]. However, both the recovery of several genomically indistinguishable clones from multiple patients, and the association between shared clonal lineages and overlapping patient admission dates is strongly suggestive, and merits future investigation. The modes of transmission and the potential reservoirs of these transmitted organisms must also be investigated through future work that more comprehensively surveys occupants of a hospital system, possibly extending to medical staff and patient family members. Regardless, it is clear that genomic analysis will be necessary to identify cryptic sharing of clonally related microbes, and to more fully illuminate the population biology of bacterial infections observed in the hospital setting.

Routine, unbiased, and large-scale sequencing of bacterial clinical isolates has the power to reveal unknown and unsuspected properties about bacterial infectious disease, and is becoming increasingly feasible with sustained advancements in massively parallel sequencing technologies. Comprehensive sequence information, derived from all bacterial isolates, has the capability to transform hospital practices not only within clinical microbiology laboratories but also by directly informing patient care in the form of infection control and treatment practices. Even when performed on a large scale, whole genome sequencing of bacterial isolates may prove cost effective in healthcare practice, considering the financial savings that would accompany potentially reduced patient morbidity and mortality. Although it will take some time to fully explore the power of this approach in the clinical identification and management of bacterial infectious disease, we have demonstrated that there are practical applications to be realized through the immediate application of large-scale, unbiased sequencing of clinical bacterial isolates.

## Materials and Methods

### Isolate collection

Primary isolation and characterization of bacterial isolates was performed by the University of Washington Medical Center Clinical Microbiology Laboratory according to routine laboratory practices [26]. Antibiotic susceptibility testing was performed using a combination of the TREK Sensititre system (TREK Diagnostic Systems) and E-test (bioMerieux SA), depending on the organism isolated, when clinically indicated. Two mycobacterial isolates (*Mycobacterium tuberculosis and Mycobacterium avium*) were excluded from the study. Primary bacterial isolates were collected from primary diagnostic culture or subculture plates, and grown overnight in liquid cultures of Luria-Bertani medium or streaked for confluence and grown as a lawn on appropriate solid media, depending on nutritional requirements. Organisms reported as part of aggregated populations (for example “mixed Gram positive flora”) were not analyzed. Use of microbiological specimens and patient chart review was approved by the University of Washington Human Subjects Review Board (approval number 42541) and was conducted in accordance with the Declaration of Helsinki. As a minimal risk study utilizing surplus microbiological isolates, a waiver of consent was approved, as it was not possible to contact all subjects associated with the isolates nor feasible to obtain consent from the study population, and the waiver of consent was not deemed to adversely affect the rights and welfare of the subjects. Results of this study research study were not directly communicated to clinical providers or used as actionable information.

### Sequencing, de novo assembly and genomic taxonomy classification

After the completion of isolate collection, sequencing libraries were constructed in serial batches of ~192 organisms each, using the same lots of reagents in order to limit batch effects. Sequencing libraries were prepared as described elsewhere [30], with the addition of a size selection step to enrich for library fragments 400–900 bp in size. Sequencing was performed using Illumina HiSeq 2000 and Illuimina MiSeq platforms with 101 bp paired end reads. Adaptors were trimmed and PCR duplicates removed using the program *Fastq-Mcf* (http://code.google.com/p/ea-utils/) with skew filtering disabled and other parameters at default. Draft genomes were assembled using *AbySS* v1.3.5 [65], with *k*-mer values empirically optimized to maximize the N50 statistic (the length for which all contigs of equal or larger size contain half the sum of the entire assembly) of assemblies (**S1 Data File**). To perform species-level classification of isolates, contigs from each isolate were BLAST [66] searched against bacterial genomes in the NCBI non-redundant nucleotide database (NT, accessed 4-15-14). The best BLAST hit (as measured by e-value) for each contig matching a partial or complete genome was recorded, and the cumulative length of best-matched contigs was recorded for each organism: the organism with the greatest cumulative length of best matched designated as the closest match. Estimated read depth per strain was calculated by the Lander-Waterman method [67], assuming the length of the closest-matched genome. Genomes with less than 7X estimated average read depth were discarded as a quality control measure.

### Identification of novel genomospecies

Average Nucleotide Identity by BLAST (ANIb) values were calculated for draft genomes using the jSpecies algorithm [27] as implemented through a standalone script (https://github.com/widdowquinn/scripts/blob/master/bioinformatics/calculate_ani.py). Isolates were considered to be from a novel genomospecies if they exhibited <95% ANIb when compared against their closest-matched genome, as identified above.

Clustering of like organisms was performed on the basis of ANIb values using an agglomerative hierarchical clustering approach, both for the entire collection of isolates and for isolates from novel genomospecies. ANIb values were first calculated for all bacterial isolates to generate an all-by-all comparison matrix. We then created a network in which each node corresponded to a bacterial isolate, and the edges between nodes were given weights equal to the isolates’ pairwise ANIb values, up to a maximum of 0.95. We next applied agglomerative hierarchical clustering as described elsewhere [31]. To determine the optimal number of clusters, which corresponds to an estimate of the number of distinct species, we calculated the intra-cluster enrichment metric across all cluster numbers from 0 to 150 (**S4** and **S5 Figs**). Individual isolates that were not initially assigned to a cluster were added to existing clusters if they matched an isolate within it with ANIb ≥ 0.95. Clustering images were generated with igraph (http://www.igraph.org/). Some isolates were left unclustered because they did not connect closely to any other isolates.

### Molecular epidemiology

Analysis was confined to isolates matching an available reference genome with an ANIb value of ≥95%. Single nucleotide variants were called by aligning short read data from sequenced strains against appropriate reference genomes using BWA version 0.6.1-r104 [68] and SAMtools version 0.1.18 [69], discarding reads with mapping quality of less than 10. To avoid artifacts relating to the inclusion of genomically disparate species, we excluded isolates qualifying as novel genomospecies by ANIb analysis. Single nucleotide variant calling was performed using SAMtools with a haploid genome model and minimum variant frequency of 0.5. Variants supported by fewer than 5 reads or a likelihood score of less than 200 were masked as “unknown” data. All-by-all pairwise distance matrices were constructed using custom perl scripts by comparing sites of variation among isolates, masking sites at which one or both isolates displayed “unknown” data or fewer than 15× read coverage, and counting only those variants sites at which both isolates could be confidently genotyped. Pairwise genomic distances were expressed as the absolute number of passing variant sites which distinguished such pairs. For focused mutational analyses, variants were annotated using snpEFF version 4.1G [70].

### Pan-genome analyses

Draft genome sequences and available complete genomes from NCBI were combined for pan-genome analysis as described elsewhere [14], with minor modifications. Briefly, gene predictions were made using RAST version 4.0 [71]. A “meta-reference” was constructed to represent all unique coding sequences (CDS) in all strains, and were clustered using CD-HIT v4.6 [72] to de-duplicate proteins ≥80% identical. Putative phage and insertion sequences were identified by BLAST search against a prophage database as described [73] and eliminated. BLASTX and BLASTP were used to search *de novo* assemblies and complete genomes, respectively, against the meta-reference, and a CDS was considered present in an isolate if ≥80% of the CDS was covered by an alignment and protein-level identity was ≥80%. Sequences of ≤75 amino acids in length and assemblies with an N50 statistic of <8x10^3^ bp were excluded from pan-genome analysis. 1,000 different random input orders of genomes were performed using the *specaccum* function in the *vegan* package implemented in R 3.1.1, and standard power functions with offsets were fit to resultant data using *pyeq2* (http://code.google.com/p/pyeq2/) to enable imputing gene diversity for different numbers of genomes sequenced.

## Supporting Information

S1 Fig Sites of isolation for bacterial isolates. Data are stratified for most frequent culture sites, with less frequent culture types aggregated as “other”. Red bars indicate the percentage of all isolates that were sampled from the indicated culture site. Blue bars indicate percentage of all isolates that qualified as novel genomospecies by ANIb analysis.
(PDF)

S2 Fig Histogram of ANIb values for sequenced isolates. (PDF)

S3 Fig Percentage of novel genomospecies in genera. All genera with >5 isolates sequenced are indicated. The x-axis indicates the proportion of each genus that consisted of novel genomospecies by ANIb analysis. The red number to the right of each bar indicates the total number of isolates sampled from each genus in this study.
(PDF)

S4 Fig Intra-cluster enrichment metric for all organisms sequenced. Intra-cluster enrichment metric across is shown for all cluster numbers from 0 to 150. The maximum value, corresponding to the most likely number of clusters, occurs at 78.
(PDF)

S5 Fig Intra-cluster enrichment metric for novel genomospecies. Intra-cluster enrichment metric across is shown for all cluster numbers from 0 to 150 (values of zero were achieved after 81). The maximum value, corresponding to the most likely number of clusters, occurs at 53.
(PDF)

S6 Fig Pairwise ANIb values of isolates in the same group and in different groups. Data are displayed as box and whisker plots for pairwise comparisons within the same group (red) or pairwise comparisons in different groups (blue). A long tail in pairwise ANIb is observed for isolates placed in the same group, reflecting the occasional connection of separate groups by intermediate nodes that are equally close to both groups.
(PDF)

S7 Fig Time and genomic distance between isolates sampling from clonal lineages. Data are shown for 17 different clonal lineages. Time “0” represents the day that the first isolate from a clonal lineage was obtained. The number of SNPs distinguishing pairs of isolates that were obtained through adjacent collections is displayed.
(PDF)

S1 Table Average pairwise SNVs for the 20 most prevalent species. (DOCX)

S2 Table Polyclonal and longitudinal intra-patient infections. (DOCX)

S3 Table Clonal lineages found in multiple patients. (DOCX)

S4 Table Mismatch repair gene mutations in *P*. *aeruginosa* clonal lineage 11. (DOCX)

S1 Data File Sample set overview. (XLSX)

S2 Data File Species classification. (XLSX)

S3 Data File All-isolate ANIb clustering assignments. (XLSM)

S4 Data File Novel genomospecies ANIb clustering assignments. (XLSM)

S5 Data File Species pangenome data. (XLSX)

S6 Data File Pairwise SNV matrices for clonal lineages involving 3 or more patients. (XLSX)
