# Correction: A Year of Infection in the Intensive Care Unit: Prospective Whole Genome Sequencing of Bacterial Clinical Isolates Reveals Cryptic Transmissions and Novel Microbiota

**DOI:** 10.1371/journal.pgen.1006724

**Published:** 2017-04-14

**Authors:** David J. Roach, Joshua N. Burton, Choli Lee, Bethany Stackhouse, Susan M. Butler-Wu, Brad T. Cookson, Jay Shendure, Stephen J. Salipante

221 of the genome taxonomic classifications described in this article have been reclassified or updated since the time of publication. Some misidentifications were due to limitations of the BLAST-based taxonomic classification scheme used in the study and others result from updates to the NCBI GenBank database and NCBI taxonomy, which integrate sequence data that were not present in public databases at the time of the authors’ original analysis. A full list relating to the 221 genome taxonomic classifications affected in this article can be found in the attached S1 File. The conclusions and results are not affected by these misclassifications.

## Supporting information

S1 File Updated NCBI GenBank taxonomic classifications. (TXT)

## References

[1] Roach DJ, Burton JN, Lee C, Stackhouse B, Butler-Wu SM, Cookson BT, et al (2015) A Year of Infection in the Intensive Care Unit: Prospective Whole Genome Sequencing of Bacterial Clinical Isolates Reveals Cryptic Transmissions and Novel Microbiota. PLoS Genet 11(7): e1005413 doi: 10.1371/journal.pgen.1005413 26230489 10.1371/journal.pgen.1005413 PMC4521703

