# Supplementary material for: A Year of Infection in the Intensive Care Unit: Prospective Whole Genome Sequencing of Bacterial Clinical Isolates Reveals Cryptic Transmissions and Novel Microbiota
Source: PLoS Genet. 2015 Jul 31;11(7):e1005413. doi: 10.1371/journal.pgen.1005413 (PMC4521703; doi:10.1371/journal.pgen.1005413)
Supplement: S1 Fig — Data are stratified for most frequent culture sites, with less frequent culture types aggregated as “other”. Red bars indicate the percentage of all isolates that were sampled from the indicated culture site. Blue bars indicate percentage of all isolates that qualified as novel genomospecies by ANIb analysis. (PDF) [file pgen.1005413.s001.pdf]

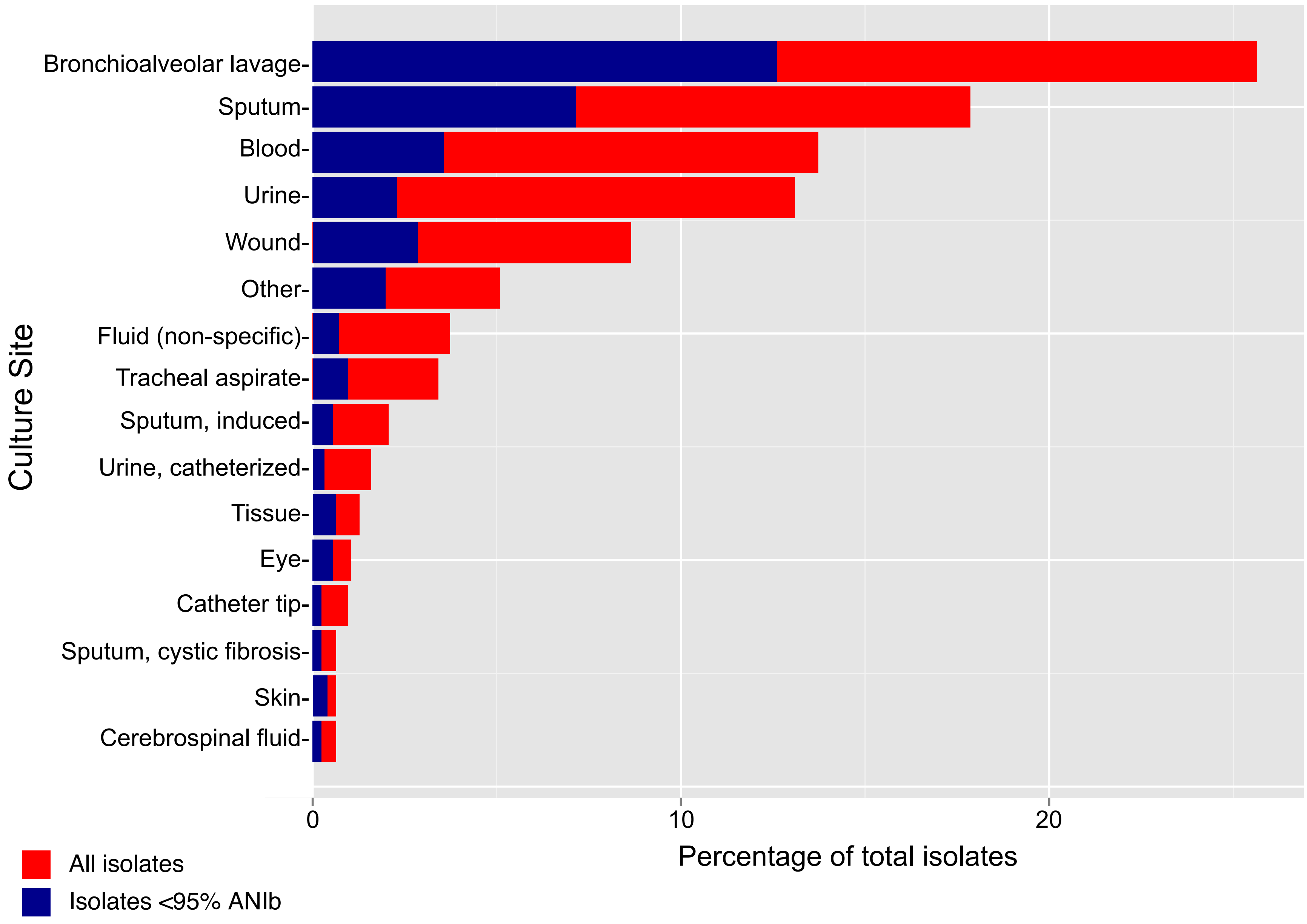
Bronchioalveolar lavage
Sputum
Blood
Urine
Wound
Other
Fluid (non-specific)
Tracheal aspirate
Sputum, induced
Urine, catheterized
Tissue
Eye
Catheter tip
Sputum, cystic fibrosis
Skin
Cerebrospinal fluid
Culture Site
0
10
20
Percentage of total isolates
All isolates
Isolates <95% ANIb
