# Supplementary figures and images for: A Year of Infection in the Intensive Care Unit: Prospective Whole Genome Sequencing of Bacterial Clinical Isolates Reveals Cryptic Transmissions and Novel Microbiota
Source: PLoS Genet. 2015 Jul 31;11(7):e1005413. doi: 10.1371/journal.pgen.1005413 (PMC4521703; doi:10.1371/journal.pgen.1005413)

ANlb Value

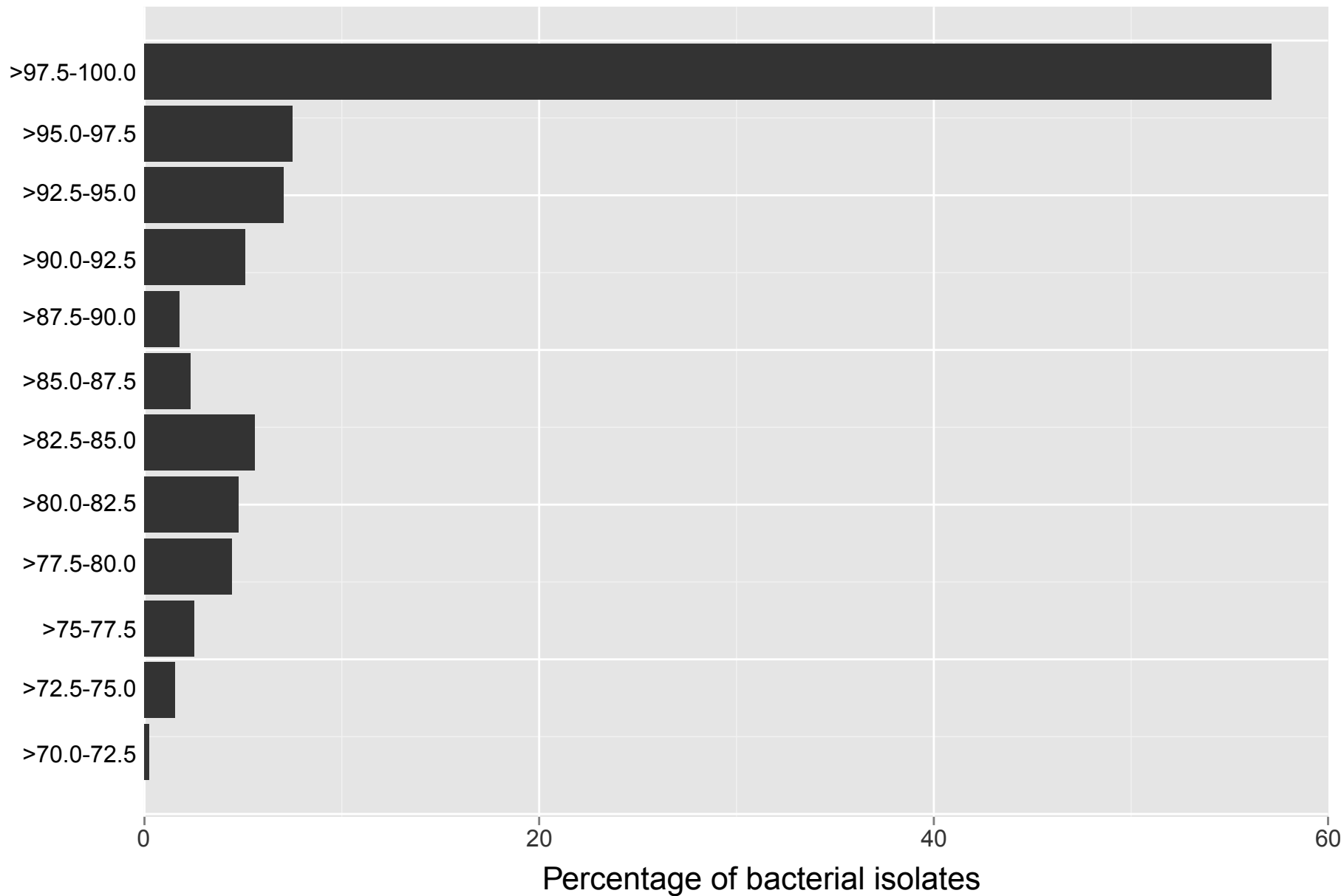

Supplement: S2 Fig — (PDF) [file pgen.1005413.s002.pdf]

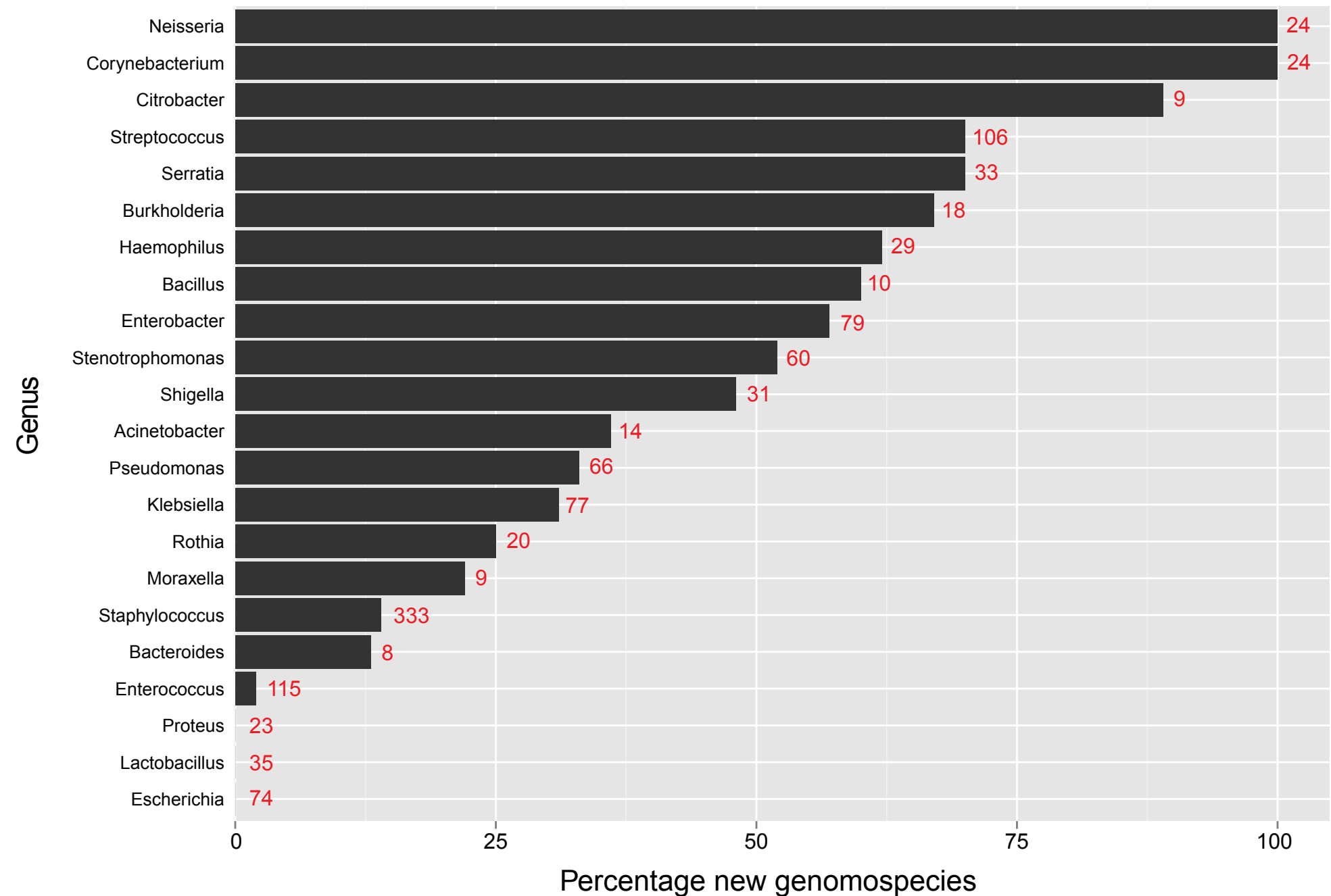

Supplement: S3 Fig — All genera with >5 isolates sequenced are indicated. The x-axis indicates the proportion of each genus that consisted of novel genomospecies by ANIb analysis. The red number to the right of each bar indicates the total number of isolates sampled from each genus in this study. (PDF) [file pgen.1005413.s003.pdf]

Enrichment Metric

300

200

100

0

0

50

100

150

Cluster Number

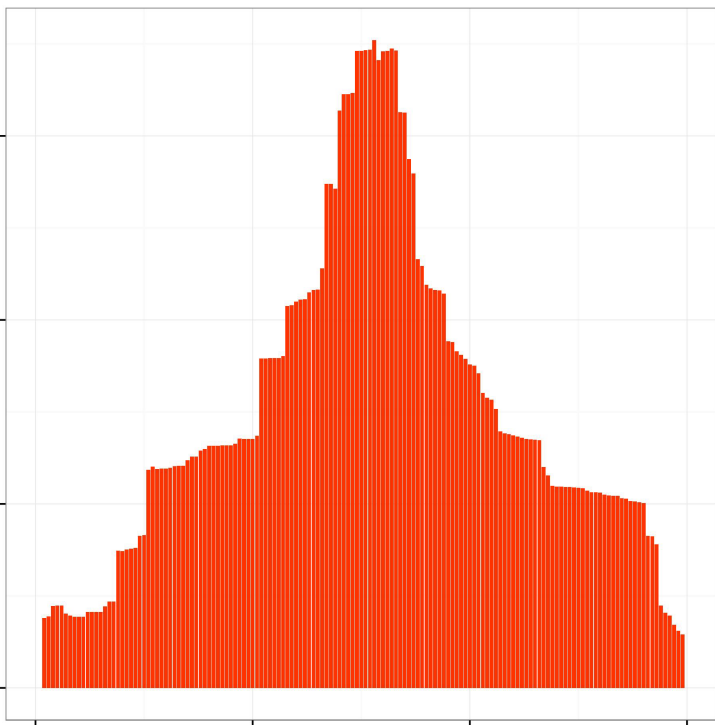

Supplement: S4 Fig — Intra-cluster enrichment metric across is shown for all cluster numbers from 0 to 150. The maximum value, corresponding to the most likely number of clusters, occurs at 78. (PDF) [file pgen.1005413.s004.pdf]

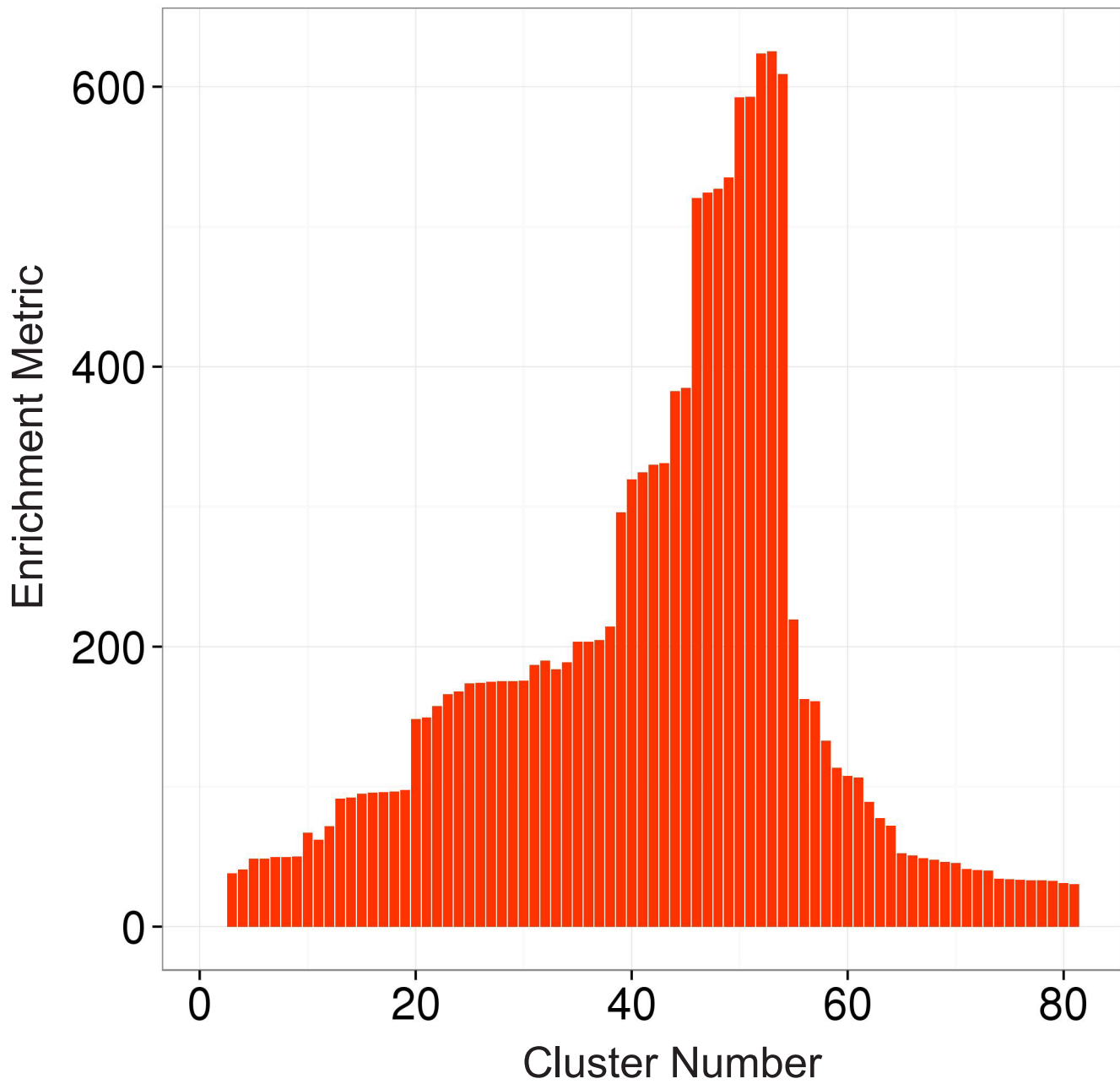

Supplement: S5 Fig — Intra-cluster enrichment metric across is shown for all cluster numbers from 0 to 150 (values of zero were achieved after 81). The maximum value, corresponding to the most likely number of clusters, occurs at 53. (PDF) [file pgen.1005413.s005.pdf]

ANlb value

1.0  
0.95  
0.75  
0.5  
0.0

Same  
Group

Different  
Groups

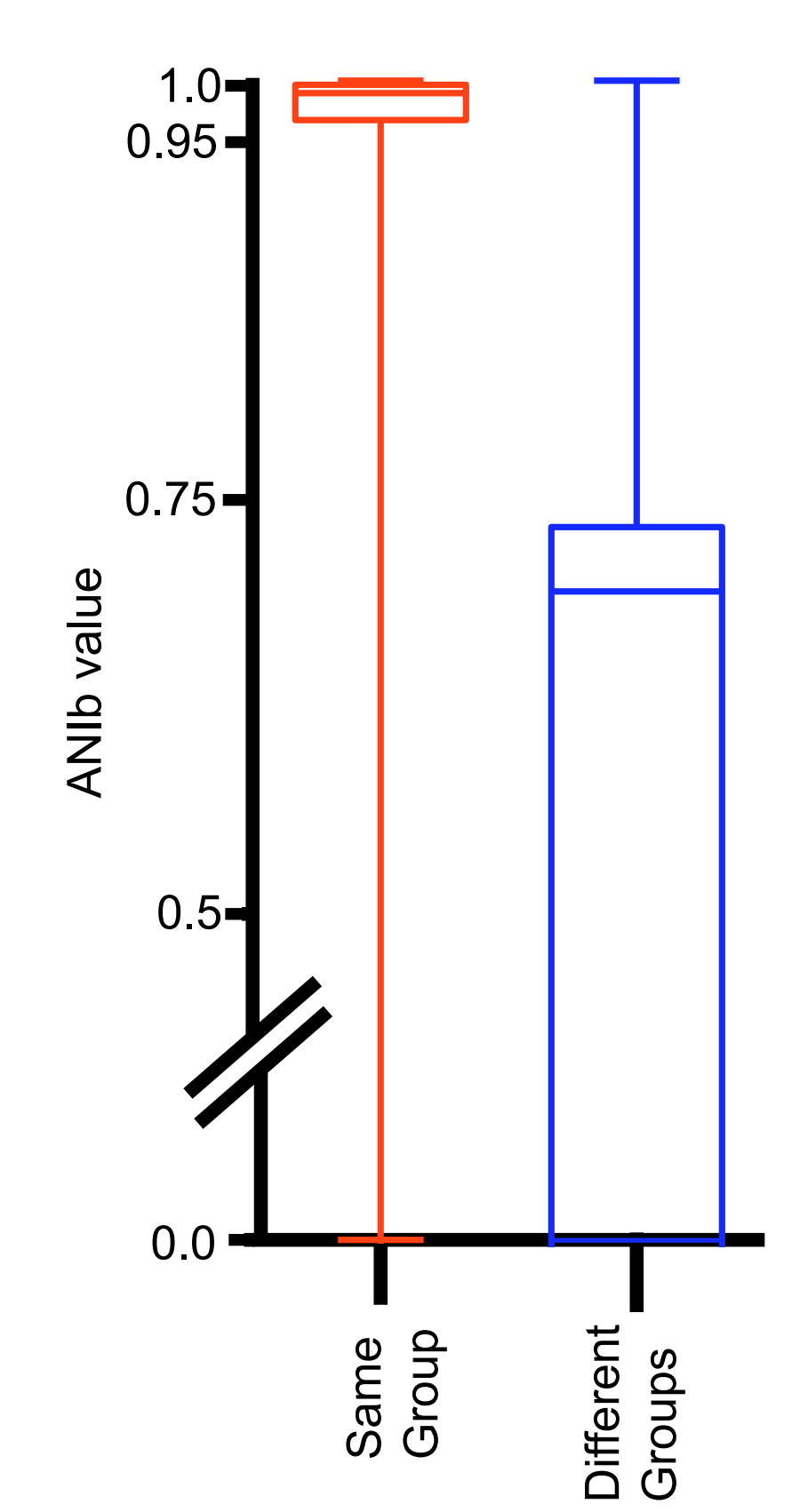

Supplement: S6 Fig — Data are displayed as box and whisker plots for pairwise comparisons within the same group (red) or pairwise comparisons in different groups (blue). A long tail in pairwise ANIb is observed for isolates placed in the same group, reflecting the occasional connection of separate groups by intermediate nodes that are equally close to both groups. (PDF) [file pgen.1005413.s006.pdf]

Number SNPs

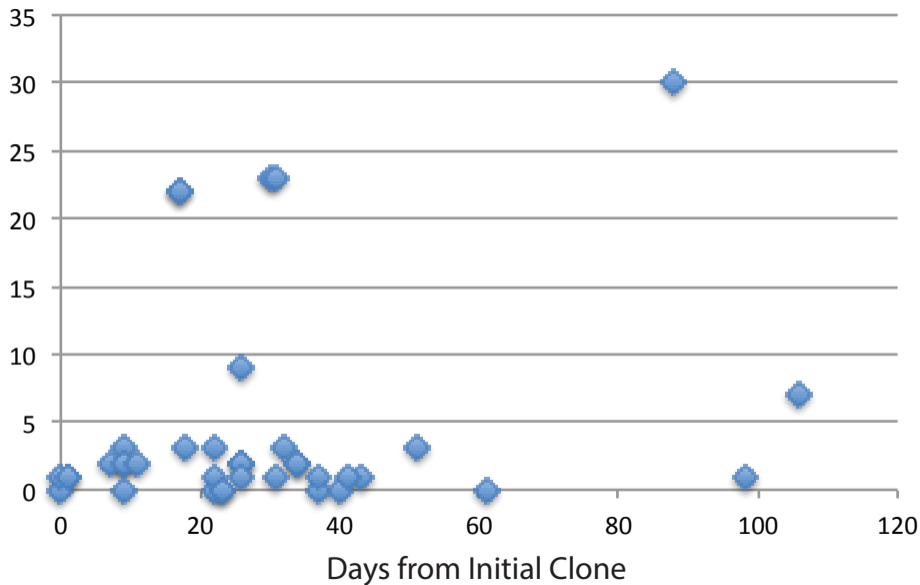

Supplement: S7 Fig — Data are shown for 17 different clonal lineages. Time “0” represents the day that the first isolate from a clonal lineage was obtained. The number of SNPs distinguishing pairs of isolates that were obtained through adjacent collections is displayed. (PDF) [file pgen.1005413.s007.pdf]
