# Supplementary material for: A Year of Infection in the Intensive Care Unit: Prospective Whole Genome Sequencing of Bacterial Clinical Isolates Reveals Cryptic Transmissions and Novel Microbiota
Source: PLoS Genet. 2015 Jul 31;11(7):e1005413. doi: 10.1371/journal.pgen.1005413 (PMC4521703; doi:10.1371/journal.pgen.1005413)
Supplement: S1 Table — (DOCX) [file pgen.1005413.s008.docx]

**Table S1- Average pairwise SNVs for the 20 most prevalent species.**

| **Organism** | **SNVs** | **StDev** | **Reference Genome GI #** |
| --- | --- | --- | --- |
| *E. faecium* | 2893.4 | 3135.9 | 529202214 |
| *S. epidermidis* | 4257.8 | 2723.8 | 57636010 |
| *E. cloacae* | 5077.9 | 6284.1 | 295095013 |
| *M. cattarhalis* | 5573.1 | 3200.8 | 295919923 |
| *S. haemolyticus* | 5623.2 | 2709 | 68445725 |
| *S. agalactiae* | 8528.1 | 2762.4 | 22535226 |
| *S. aureus* | 14701.7 | 10252.8 | 545580417 |
| *E. faecalis* | 15164.3 | 5731.9 | 397335222 |
| *P. mirabilis* | 15292.5 | 4854.9 | 172046403 |
| *A. baumanii* | 17552.3 | 7867.7 | 586958174 |
| *K. pneumoniae* | 23040.8 | 17570.3 | 549815675 |
| *P. aeruginosa* | 23637.5 | 11963.6 | 575870901 |
| *H. influenzae* | 23787.1 | 5606.2 | 317449542 |
| *S. parasanguinis* | 24125.3 | 4240.7 | 335369081 |
| *L. rhamnosus* | 25989.8 | 16097.1 | 257146922 |
| *R. mucilaginosa* | 27660.5 | 8456.6 | 283133067 |
| *K. oxytoca* | 29002.3 | 17298.4 | 365906294 |
| *E. coli* | 30262.3 | 22039.1 | 85674274 |
| *S. boydii* | 31259.7 | 23158.9 | 187427012 |
| *S. maltophila* | 35091.9 | 13962.1 | 190010013 |
| *E. aerogenes* | 37777.4 | 31078.7 | 334732565 |
| *S. marascens* | 75380.6 | 19773.4 | 573008719 |
