# Supplementary material for: A Year of Infection in the Intensive Care Unit: Prospective Whole Genome Sequencing of Bacterial Clinical Isolates Reveals Cryptic Transmissions and Novel Microbiota
Source: PLoS Genet. 2015 Jul 31;11(7):e1005413. doi: 10.1371/journal.pgen.1005413 (PMC4521703; doi:10.1371/journal.pgen.1005413)
Supplement: S2 Table — (DOCX) [file pgen.1005413.s009.docx]

**Table S2. Polyclonal and longitudinal intra-patient infections.**

| **Species** | **Polyclonal colonizations in patients** | **Longitudinal samplings of clonal bacteria** |
| --- | --- | --- |
| *Acinetobacter baumannii* | 0 | 1 |
| *Enterobacter cloacae* | 1 | 1 |
| *Enterococcus faecalis* | 2 | 2 |
| *Enterococcus faecium* | 2 | 0 |
| *Escherichia coli* | 1 | 0 |
| *Haemophilus influenzae* | 1 | 0 |
| *Klebsiella pneumoniae* | 1 | 2 |
| *Lactobacillus rhamnosus* | 2 | 0 |
| *Pseudomonas aeruginosa* | 2 | 3 |
| *Rothia mucilaginosa* | 1 | 1 |
| *Staphylococcus aureus* | 2 | 3 |
| *Staphylococcus epidermidis* | 14 | 3 |
| *Stenotrophomonas maltophilia* | 2 | 1 |
