# Supplementary material for: A Year of Infection in the Intensive Care Unit: Prospective Whole Genome Sequencing of Bacterial Clinical Isolates Reveals Cryptic Transmissions and Novel Microbiota
Source: PLoS Genet. 2015 Jul 31;11(7):e1005413. doi: 10.1371/journal.pgen.1005413 (PMC4521703; doi:10.1371/journal.pgen.1005413)
Supplement: S3 Table — (DOCX) [file pgen.1005413.s010.docx]

**Table S3. Clonal lineages found in multiple patients.**

| **Organism** | **Acquisitions** | **Clones involved** | **Clones in ≥3 patients** | **# clones with ≤40 SNVs*** | **# clones with ≤3 SNVs*** |
| --- | --- | --- | --- | --- | --- |
| *S. epidermidis* | 57 | 10 | 5 | 3 | 2 |
| *E. faecium* | 13 | 3 | 2 | 1 | 0 |
| *E. faecalis* | 6 | 3 | 0 | 1 | 2 |
| *S. aureus* | 4 | 2 | 0 | 1 | 1 |
| *P. aeruginosa* | 7 | 3 | 1 | 2 | 0 |
| *S. maltophila* | 2 | 1 | 0 | 0 | 1 |
|  |  |  |  |  |  |
| **Excluding clonal lineages identified in three or more patients.* | | | | |  |
