# Supplementary material for: A Year of Infection in the Intensive Care Unit: Prospective Whole Genome Sequencing of Bacterial Clinical Isolates Reveals Cryptic Transmissions and Novel Microbiota
Source: PLoS Genet. 2015 Jul 31;11(7):e1005413. doi: 10.1371/journal.pgen.1005413 (PMC4521703; doi:10.1371/journal.pgen.1005413)
Supplement: S4 Table — (DOCX) [file pgen.1005413.s011.docx]

**Table S4. Mismatch repair gene mutations in *P. aeruginosa* clonal lineage 11.**

|  |  |  |  |  |  | Isolate | | |
| --- | --- | --- | --- | --- | --- | --- | --- | --- |
| Position in PAO1 Genome (GI:110645304) | Reference Base | Variant Base | Gene | Coding Sequence Change | Amino Acid Change | 279_PAER | 852_PAER | 875_PAER |
| 5550510 | G | A | mutL | c.1172C>T | p.Ala391Val | + | + | ND* |
| 5551672 | C | T | mutL | c.10G>A | p.Ala4Thr | + | + | + |
| 5797013 | G | A | mutY | c.1060G>A | p.Ala354Thr | + | + | + |
| 6132753 | G | A | uvrD | c.1666G>A | p.Val556Ile | + | + | + |
| 6133008 | G | T | uvrD | c.1921G>T | p.Ala641Ser | + | + | ND |
| 6133072 | G | A | uvrD | c.1985G>A | p.Ser662Asn | + | + | ND |
| 6133084 | A | G | uvrD | c.1997A>G | p.Asn666Ser | + | + | + |

*Insufficient read depth to accurately genotype
